# Impact of COVID-19 on Universal Tumor Screening, Referral Rates and Attendance at Cancer Genetic Counseling at a Safety-Net University Hospital

**DOI:** 10.3390/curroncol32100549

**Published:** 2025-09-30

**Authors:** Dimitrios N. Varvoglis, Kelsey R. Landrum, Lydia H. Comer, Julianne M. O’Daniel, Chris B. Agala, Lacey M. Lee, José G. Guillem

**Affiliations:** 1 Department of Surgery, University of North Carolina at Chapel Hill, Chapel Hill, NC 27599, USA; 2 Department of Surgery, University of Colorado Anschutz Medical Campus, Aurora, CO 80045, USA; 3 Department of Genetics, University of North Carolina at Chapel Hill, Chapel Hill, NC 27599, USA; 4 Department of Pharmacy, University of North Carolina Health Care, Chapel Hill, NC 27599, USA; lacey.lee@unchealth.unc.edu; 5 Department of Surgery, Penn State University College of Medicine, Hershey, PA 17033, USA

**Keywords:** universal tumor screening, Lynch syndrome, pandemic, genetic counseling

## Abstract

**Simple Summary:**

In order for a genetic cause behind colorectal cancer (CRC) to be identified, a lot of healthcare systems have implemented universal tumor screening (UTS). UTS is an approach according to which every CRC is tested in order to identify those patients who are more probable to have genetics as the cause of their disease. Those patients should then be referred for further evaluation by genetic counsellors (GCs). It is known that the pandemic caused unprecedented stress to healthcare systems, but to this day, it remains unclear how the COVID-19 pandemic affected UTS. For this reason, we decided to perform this study in the hope that we will identify areas of weakness and opportunities for improvement of UTS. Our results show that, in our institution, several components of UTS work well, while others, such as the referral and attendance of patients to meetings with GCs, have room for improvement. We believe that improvement in these areas could be achieved by the earlier involvement of GCs in the process of identifying patients that may benefit from genetic evaluation.

**Abstract:**

Universal tumor screening (UTS) of all newly diagnosed colorectal cancers (CRCs) for the identification of Lynch syndrome (LS) is recommended. We explored the impact of the COVID-19 pandemic on the UTS process in a safety-net university hospital to identify areas of vulnerability and opportunities for improvement. Patients undergoing resection of a primary CRC were categorized into three cohorts based on surgery date relative to the pandemic (pre-[2018,2019], early-[2020,2021] and late-[2022]). Data regarding (1) UTS performance of immunohistochemistry (IHC) for LS genes and microsatellite instability (MSI) testing; (2) referrals to cancer genetic counseling (CGC) based on mismatch repair deficient (dMMR) status and/or age < 50 years at diagnosis; (3) attendance at CGC; and (4) reasons for not attending CGC were extracted. Between 2018 and 2022, 342 patients underwent resection of a CRC. During the three time periods (pre-, early- and late-pandemic), 93%, 94% and 96% of cases were screened with at least MMR IHC, respectively. Of the patients eligible for referral to CGC in each time period, 60%, 71% and 63% had a referral submitted. Of these, 23%, 36% and 20% in each time period did not attend CGC, with the most common reason for not attending being the inability of schedulers to reach the patient. Although the COVID-19 pandemic did not cause significant variation in the different steps of the UTS process, CGC utilization remained suboptimal throughout the three time periods. Further research on barriers preventing physicians from referring patients to CGC as well as schedulers inability to reach eligible patients should be pursued.

## 1. Introduction

Colorectal cancer (CRC) is the third most commonly diagnosed cancer in the United States, with multiple factors including diet, obesity, inflammatory bowel disease and family history of CRC increasing the risk of developing the disease [1]. Lynch syndrome (LS) which constitutes the most common form of hereditary CRC and accounts for approximately 3% of the total CRC burden [2,3], is inherited in an autosomal dominant fashion with mutations most commonly found in the mismatch repair genes (MMRs), namely *MLH1*, *MSH2*, *MSH6* and *PMS2* [4,5]. In order to implement early screening, chemoprevention and possible risk-reducing surgeries, the identification of patients and families at risk of developing inherited CRC, such as LS, is essential [6,7,8,9].

In 2009, the concept of universal tumor screening (UTS) of every newly diagnosed CRC was introduced [10]. Although UTS, which consists of immunohistochemistry (IHC) analysis for LS-associated gene expression and/or microsatellite instability (MSI) testing, has been endorsed by multiple societies and is relatively inexpensive, its uptake remains a challenge [11,12,13,14,15].

Given that the COVID-19 pandemic caused disruption in various steps of the CRC care continuum, including screening [16] and treatment [17], the impact of the pandemic on UTS has, to the best of our knowledge, not been previously examined. Therefore, our study explored the potential impact of the COVID-19 pandemic on the multiple steps of the UTS process in CRC patients in a safety-net university hospital to identify areas of vulnerability and opportunities for improvement in the implementation of UTS.

## 2. Methods

Using departmental billing records, patients with a new diagnosis of colon and/or rectal adenocarcinoma who underwent resection at the University of North Carolina (UNC) Medical Center in Chapel Hill between 2018 and 2022 were identified. UNC Medical Center is recognized as a safety-net hospital because it is committed to delivering care to the uninsured, underinsured and/or Medicaid beneficiaries. Two independent reviewers (DNV, LHC) reviewed electronic medical records (EMRs) and extracted patient data on the following: (1) age at diagnosis; (2) gender; (3) race; (4) primary diagnosis; (5) year of primary tumor resection; (6) performance and results of immunohistochemistry (IHC) for LS genes; (7) performance and results of microsatellite instability (MSI) testing; (8) performance of MLH1-promoter hypermethylation testing, when indicated; (9) appropriate referral to CGC; (10) attendance at CGC, if referred; and (11) reason(s) for not attending CGC. Given that a predetermined timeframe for CGC attendance did not exist, a thorough search of the EMR that included the extraction of data, if present, from the “Genetics” “Activity Tab” of Epic Hyperspace and the “Genetics Notes” found in the “Chart Review” “Activity Tab” of Epic Hyperspace was undertaken. If the aforementioned “Activity Tabs” did not contain any extractable information, a search using the broad keyword “genetic” was performed. This advanced search facilitated the capture of all CGC attendances, including those occurring outside UNC Medical Center.

Based on the year of primary tumor resection relative to the COVID-19 pandemic, three cohorts were created, namely pre-(2018–2019), early-(2020–2021) and late-(2022). This grouping allowed for the creation of three distinct cohorts representative of three distinct periods of UTS performance relative to the COVID-19 pandemic: (1) “pre-pandemic” cohort to be representative of the baseline UTS performance before the pandemic; (2) “early-pandemic” cohort to be representative of the performance of the UTS while strict pandemic measures were still in place; and (3) “late-pandemic” cohort to be representative of the UTS performance when the majority of restriction measures were alleviated and vaccines were widely available. Patients were excluded if they (1) did not undergo resection of their primary tumor at UNC Medical Center in Chapel Hill; (2) did not have a pathological diagnosis of adenocarcinoma and (3) underwent resection of their primary tumor before the time period of interest. Eligibility for a referral to CGC was based upon the following: (1) abnormal IHC and/or MSI results (dMMR); (2) lack of *MLH1* promoter hypermethylation, if *MLH1* loss was detected; (3) EO CRC, defined as age < 50 years at diagnosis; and (4) absence of a known history of a hereditary cancer syndrome. According to the institutional protocol, schedulers were expected to make 3 attempts to reach referred patients. If patients could not be reached, this was documented in the patient’s EMR. Data on the performance rate of UTS, namely IHC and/or MSI testing, referral rate to CGC when indicated and attendance rates at CGC for the three study cohorts, pre-, early- and late-pandemic, were compared.

## 3. Statistical Analysis

Univariate statistics were used to describe the following: (1) primary diagnosis (rectal or colon cancer); (2) age; (3) EO (yes [<50 years], no [≥50 years]); (3) sex assigned at birth (male, female); (4) self-reported race (White, African American, American Indian/Alaskan Native, Asian, Other). The chi-square test of independence and the Wilcoxon rank–sum test were used to test categorical and continuous baseline characteristics for their potential to predict each outcome.

Outcomes were reported using univariate statistics, followed by pairwise unadjusted comparisons of each outcome (pre- vs. early-pandemic; pre- vs. late-pandemic; early- vs. late-pandemic) using chi-square test of independence and Wilcoxon rank-sum test for categorical and continuous covariates, respectively. Poisson regression (log link, Poisson distribution) with robust standard errors (SEs) to adjust for EO status, MMR status, sex, race and estimate risk, risk rations (RRs) with corresponding 95% confidence intervals (CIs) as well as *p*-values (alpha = 0.05) for each outcome was used [18,19]. Poisson regression was preferred over traditional log-risk regression to avoid convergence issues [18,19].

Additional subgroup analyses on patients that (1) were tested with IHC; (2) had abnormal IHC; (3) received a referral to CGC, when indicated and (4) attended CGC after being referred were performed. The analysis was performed in SAS 9.4 for Windows [20].

## 4. Results

A total of 495 patients with CRC were identified; of these, 153 were excluded for reasons stated above. Our study population therefore comprised 342 patients: 152 in the pre-pandemic, 139 in the early-pandemic and 51 in the late-pandemic cohort.

### 4.1. Cohort Descriptions

#### 4.1.1. Pre-Pandemic Cohort

A total of 152 patients with a mean age of 61.3 years underwent resection of their primary CRC at UNC Medical Center in Chapel Hill between 2018 and 2019. Of these, 46% were female and 73%, 16% and 11% were, as recorded in EMR, White, African American and Asian/American Indian/Alaska Native, respectively. Approximately 22% were younger than 50 years of age at diagnosis and 54% were diagnosed with rectal adenocarcinoma (Table 1).

Ninety-three percent (93%) of the tumors were screened with at least MMR IHC for loss of expression of LS-related genes (Table 2). The majority (94%, n = 133/141) of those tested with IHC were MMR proficient (pMMR) with only 6% (n = 8/141) dMMR. Sixty-two percent (62%) of the dMMR tumors demonstrated a loss of both *MLH1* and *PMS2*, and, of these, sixty percent (60%) tested positive for *MLH1* promoter hypermethylation. Loss of *MSH2* or combined *MSH2*/*MSH6* expression was seen in 25% and 13% of dMMR tumors, respectively (Table 3). In total, 63% (n = 5/8) of the dMMR were suspicious for LS based on IHC.

A total of 37 (24%) of the pre-pandemic cohort patients were eligible for a referral to CGC based on the previously described criteria. Of these, 60% had a referral to CGC placed in the EMR, and of those referred, 77% attended CGC (Table 4). The most common reason for a patient to not attend CGC, if referred, was the inability of schedulers to reach them, despite three attempts per institutional protocol (Table 5). Of note, only 46% (n = 17/37) of the patients eligible for CGC were referred to and actually attended CGC in the pre-pandemic cohort.

#### 4.1.2. Early-Pandemic Cohort

A total of 139 patients with a mean age of 61.7 years underwent resection of their primary CRC at UNC Medical Center in Chapel Hill during 2020 and 2021. Of these, 42% were female and 72%, 23% and 5% were, as recorded in EMR, White, African American and Asian/American Indian/Alaska Native, respectively. Approximately 19% were younger than 50 years of age at diagnosis and 43% were diagnosed with rectal adenocarcinoma (Table 1).

Ninety-four percent (94%) of the tumors were screened with at least MMR IHC for loss of expression of LS-related genes (Table 2). The majority (84%, n = 109/130) were pMMR with only 16% (n = 21/130) dMMR. Sixty-seven percent (67%) of the dMMR tumors demonstrated a loss of both *MLH1* and *PMS2*, and of these, fifty-seven percent (57%) were *MLH1* promoter hypermethylation positive, while only two (14%) were *MLH1* promoter hypermethylation negative. A total of four patients (29%) were not tested for *MLH1* promoter hypermethylation, despite abnormal IHC results. Loss of *PMS2*, *MSH2*, *MSH6* or combined *MSH2*/*MSH6* was seen in 9%, 5%, 5%, and 14% of the dMMR tumors, respectively (Table 3). In total, 62% (n = 13/21) were suspicious for LS based on IHC.

A total of 35 (25%) of the early-pandemic cohort patients were eligible for CGC referral based on the previously described criteria. Of these, 71% had a referral placed in the EMR, and of those referred, 64% attended CGC (Table 4). The most common reasons for a patient to not attend CGC were the inability of schedulers to reach them, despite three attempts per institutional protocol and the fact that some patients declined the opportunity to receive CGC and/or genetic testing (Table 5). Of note, only 46% (n = 16/35) of the patients eligible for CGC were referred to and actually attended CGC in the early-pandemic cohort.

#### 4.1.3. Late-Pandemic Cohort

A total of 51 patients with a mean age of 62.5 years underwent resection of their primary CRC at UNC Medical Center in Chapel Hill during 2022. Of these, 43% were female and 75%, 16% and 9% were, as recorded in EMR, White, African American and Asian/American Indian/Alaska Native, respectively. Approximately 12% were younger than 50 years of age at diagnosis and 57% were diagnosed with rectal adenocarcinoma (Table 1).

Ninety-six percent (96%) of the tumors were screened with at least MMR IHC for loss of expression of LS-related genes (Table 2). The majority (88%, n = 43/49) were pMMR and 12% (n = 6/49) dMMR. Eighty-three percent (83%) of the dMMR tumors demonstrated a loss of both *MLH1* and *PMS2* and, of these, one hundred percent (100%) were *MLH1* promoter hypermethylation positive. Loss of *MSH6* was noted in 17% (Table 3). In total, 17% (n = 1/6) were suspicious for LS based on IHC.

A total of eight (16%) of the late-pandemic cohort patients were eligible for a CGC referral based on the previously described criteria. Of these, 63% had a referral placed in the EMR and, of those referred, 80% attended CGC. (Table 4) The most common reason for a patient to not attend CGC was the inability of schedulers to reach them, despite three attempts per institutional protocol (Table 5). Of note, only 50% (n = 4/8) of the patients eligible for CGC were referred to and actually attended CGC in the late-pandemic cohort.

### 4.2. Comparison of Baseline Cohort Characteristics (Table 1)

Pairwise comparisons of patient baseline characteristics demonstrated no differences across the groups in terms of age, EO status, sex, race or diagnosis.

### 4.3. Comparison of Universal Tumor Screening Performance (Table 2)

Pairwise comparisons of the percentage of CRCs that were screened with at least MMR IHC and/or MSI testing indicated no significant differences across the three cohorts (early- vs. pre-pandemic, *p* = 0.80; late- vs. pre-pandemic, *p* = 0.40; late- vs. early-pandemic, *p* = 0.50).

### 4.4. Comparison of the Immunohistochemistry Results

Pairwise comparisons of the percentages of dMMR and pMMR between the different cohorts revealed that in the early-pandemic cohort a higher proportion of tumors were dMMR compared with the pre-pandemic cohort (16.2% [n = 21/130] vs. 5.7% [n = 8/141], *p* = 0.005). No significant differences were noted when comparing the late-pandemic with the pre-pandemic cohort (*p* = 0.13) and the late-pandemic with the early-pandemic cohort (*p* = 0.52). No differences were noted when comparing the frequencies of suspicious and sporadic tumors across the three cohorts (early- vs. pre-pandemic, *p* = 0.98; late- vs. pre-pandemic, *p* = 0.09; late- vs. early-pandemic, *p* = 0.05).

### 4.5. Comparison of the Eligible Patient Percentages for Cancer Genetic Counseling Referral (Table 4)

Pairwise comparisons of the percentages of eligible patients for CGC referral did not differ among the three cohorts (early- vs. pre-pandemic, *p* = 0.87; late- vs. pre-pandemic, *p* = 0.20; late- vs. early-pandemic, *p* = 0.17).

### 4.6. Comparison of Referral Rates of Eligible Patients to Cancer Genetic Counseling and Predictors of Having a Referral Placed (Table 4 and Table A1)

Pairwise comparisons of the percentages of eligible patients who received referral to CGC across the three cohorts did not demonstrate any significant differences (pre- vs. early-pandemic, *p* = 0.29; pre- vs. late-pandemic, *p* = 0.87; early- vs. late-pandemic, *p* = 0.62). Additionally, there were no factors predictive in any of the three cohorts of who would have a referral to CGC in place.

### 4.7. Comparison of Attendance Rates of Eligible and Referred Patients to Cancer Genetic Counseling and Predictors of Attending (Table 4 and Table A2)

Pairwise comparisons of the percentages of eligible patients for CGC who were referred and attended an appointment did not demonstrate any significant differences across the three cohorts (pre- vs. early-pandemic, *p* = 0.32; pre- vs. late-pandemic, *p* = 0.90; early- vs. late-pandemic, *p* = 0.50). Additionally, there were no factors predictive in any of the three cohorts of who would attend CGC if a referral had been placed.

## 5. Discussion

Our study, which examined the multiple steps of the UTS-based process for the identification of LS patients in a safety-net university hospital, demonstrates that, although UTS may be performed at high rates, the subsequent necessary components of this process, namely referral and attendance at CGC, appear to have been performed at disproportionally inferior rates. However, the overall performance of the UTS-based process did not appear to be influenced by the COVID-19 pandemic.

The first step of the UTS process, namely IHC assessment for MMR protein expression and/or MSI, was consistently performed (93–96%) in the three study cohorts. These results, which align with recent reports [12,21,22,23,24,25], confirm that screening of all CRCs with MMR IHC and/or MSI regardless of patient age and family history can be routinely implemented. Notably, the completion rate of the first step in our institution was higher than previously reported national and international pooled rates [26,27,28]. In fact, a recent meta-analysis indicated that the first step of the process was completed at a rate of approximately 88%, while a recent analysis of data coming from three Danish national registries demonstrated that approximately 89% of all CRCs are screened with IHC and/or MSI throughout Denmark [26,28]. This fact, along with the high performance of this step despite the COVID-19 pandemic suggests that it may be a streamlined process at the institutional level. Of interest, although the percentage of dMMR tumors was greater in the “early-pandemic” cohort compared with the “pre-pandemic” cohort, this can perhaps be explained by the relatively larger number of sporadic MLH1 tumors identified in the “early-pandemic” cohort. Additionally, although not statistically significant, the mean age in the “early-pandemic” cohort was slightly older than that noted in the “pre-pandemic” cohort.

The second step of the UTS process, namely accurate identification and referral of eligible patients to CGC, was unfortunately less frequently (60–70%) performed. Similar to the first step, the COVID-19 pandemic did not appear to impact step two of the UTS process. Although our rates of referral to CGC were slightly higher than previously reported [12,13,26,29,30,31,32], they were nevertheless suboptimal since one in three of our eligible patients was not appropriately referred to CGC. These suboptimal referral rates may be due to the UNC Chapel Hill Medical Center UTS protocol at that time which relied solely on the genetic counselor (GC) review of MSI and MLH1 methylation results to identify an indication for CGC referral. When an indication was identified, the primary surgeon would be prompted via an EMR message to place a referral to CGC. However, in our institutional experience, 23% of patients with CRC did not have MSI performed and therefore it is possible that a fraction of patients may not have been reviewed by a GC, leading to missed opportunities for CGC referral.

The third step of the institutional UTS process demonstrated that the attendance of eligible patients referred to CGC, despite the availability of telehealth since 2020, ranged between 65% and 80%, which aligns with previous studies [29,33,34]. As previously reported [35], the most common reasons for not attending CGC were the inability of schedulers to reach patients, based on the standard health system protocol for three attempts and the fact that some patients declined the opportunity to receive CGC and/or genetic testing. A strategy to overcome these barriers could include assuring the availability of a GC during the first postoperative visit. This in-person interaction would facilitate a rapport between the GC and the patient and potentially alleviate patient-based reservations and increase acceptance to undergo genetic counseling and testing [36,37,38]. However, given the cost associated with having GC readily available, another strategy that could potentially improve the attendance rates for CGC would include the establishment of point-of-care protocols. According to those protocols, a GC would review cases during the surgical tumor board and help identify those patients that should be referred to CGC. This strategy may be more feasible, especially in systems lacking an abundance of GCs.

Our study has several limitations. First of all, it was a single institution retrospective review of EMR data. Secondly, the sample size was relatively small. Thirdly, since we did not have access to data regarding family cancer history, we were unable to assess the potential impact of a positive family history of LS-related cancers on CGC referrals. Lastly, we were not able to identify factors to account for the low referral rates or barriers preventing patients from attending CGC. It is possible that a lack of standardized roles and responsibilities within a multidisciplinary team along with a lack of awareness of current UTS guidelines may have hindered the referral of eligible patients to CGC [36,39]. In fact, previous qualitative research has demonstrated that a clear delineation of responsibilities and active participation by GCs in the identification and referral process can improve the referral rates to CGC [40]. Similarly, a recent study implementing a UTS protocol whereby GCs followed up with the treating physician when a referral had not been placed after a predetermined time period, demonstrated a 92% referral rate compared with 28% without GC involvement [33]. Lastly, another study emphasizing the added value of GCs in the UTS process demonstrated that allowing GCs to independently submit the CGC referrals resulted in referral rates of 100% [34].

## 6. Conclusions

Overall, our study uniquely demonstrates that the identification of patients with a genetic predisposition to CRC via a UTS program is feasible at a safety-net university hospital. Although the COVID-19 pandemic did not appear to affect the overall performance of the UTS program, our results highlight opportunities for improvements in CGC referral and attendance. We believe that further improvement in the UTS program may be achieved with the earlier participation of GCs in the identification of patients with CRC suitable for CGC. In addition, qualitative interviews with members of the multidisciplinary care team may provide further insights into barriers to CGC referral.

## Figures and Tables

**Table 1:** Patient baseline characteristics.

**Characteristic**	**Frequency**								**Pairwise Comparisons**		
	**Overall Cohort**		**Pre-Pandemic (2018–2019)**		**Early-Pandemic (2020–2021)**		**Late-Pandemic (2022)**		**Early- vs. Pre-Pandemic (Ref)**	**Late- vs. Pre-Pandemic (Ref)**	**Late- vs. Early- Pandemic (Ref)**
	**n**	**%**	**n**	**%**	**n**	**%**	**n**	**%**	***p*-Value**		
	342	100	152	44	139	41	51	15			
**^1^ Age in Years Mean**	61.6		61.3		61.7		62.5		0.75	0.51	0.68
SD	13.4		13.8		13.6		11.7				
**^2^ EO**	66	19	34	22	26	19	6	12	0.44	0.10	0.26
**^2^ Sex**									0.54	0.72	0.93
Male	191	56	82	54	80	58	29	57			
Female	151	44	70	46	59	42	22	43			
**^2^ Race**									0.07	0.96	0.31
White	249	73	111	73	100	72	38	75			
African American	64	19	24	16	32	23	8	16			
^3^ Other	29	8	17	11	7	5	5	9			
**^2^ Diagnosis**									0.07	0.72	0.09
Colon Cancer	171	50	70	46	79	57	22	43			
Rectal Cancer	171	50	82	54	60	43	29	57			

SD—standard deviation. EO status—early age of onset colorectal cancer status, defined as age < 50 years at diagnosis. Ref—reference group, ^1^ tested with Wilcoxon rank-sum test, ^2^ tested with chi-square test of independence; alpha = 0.05, ^3^ Asian, American Indian/Alaska Native, Other

**Table 2:** Trends of universal tumor screening at UNC pre-, early- and late-COVID-19.

	Frequency								Pairwise Comparisons		
	Overall Cohort		Pre-Pandemic (2018–2019)		Early-Pandemic (2020–2021)		Late-Pandemic (2022)		Early- vs. Pre-Pandemic (Ref)	Late- vs. Pre-Pandemic (Ref)	Late- vs. Early- Pandemic (Ref)
	n	%	n	%	n	%	n	%	*p*-Value		
	342	100	152	44	139	41	51	15			
^1^ Tested with both IHC and MSI	241	71	110	72	95	69	36	71	0.45	0.81	0.77
^1^ Tested with IHC but not MSI because of ITS	35	10	23	15	10	7	2	4	0.03 *	0.04 *	0.41
^1^ Tested with IHC and not MSI	44	13	8	6	25	18	11	21	0.001 *	0.001 *	0.58
^1^ Tested with neither IHC nor MSI	22	6	11	7	9	6	2	4	0.80	0.40	0.50
^1^ Tested with either IHC and/or MSI	320	94	141	93	130	94	49	96	0.80	0.40	0.50

IHC—immunohistochemistry; MSI—microsatellite instability; ITS—insufficient tumor sample; CGC—cancer genetic counseling. Ref—reference, ^1^ tested with chi-square test of independence; alpha = 0.05, * indicates statistical significance

**Table 3:** Proteins lost in immunohistochemistry of patients with mismatch repair deficient tumors.

	Pre-Pandemic (2018/2019) (n = 8)	Early-Pandemic (2020/2021) (n = 21)	Late-Pandemic (2022) (n = 6)
dMMR IHC	– 25% (n = 2/8) *MSH2* – 13% (n = 1/8) *MSH2* & *MSH6* – 62% (n = 5/8) *MLH1* & *PMS2* 60% (n = 3/5) *MLH1*-p-Hm (+) 40% (n = 2/5) *MLH1*-p-Hm (−)	– 5% (n = 1/21) *MSH2* – 14% (n = 3/21) *MSH2* & *MSH6* – 5% (n = 1/21) *MSH6* – 48% (n = 10/21) *MLH1* & *PMS2* 50% (n = 5/10) *MLH1*-p-Hm (+) 20% (n = 2/10) *MLH1*-p-Hm (−) 30% (n = 3/10) *MLH1*-p-Hm not tested – 19% (n = 4/21) *MLH1* (weak) & *PMS2* 75% (n = 3/4) *MLH1*-p-Hm (+) 25% (n = 1/4) *MLH1*-p-Hm not tested – 9% (n = 2/21) *PMS2*	– 17% (n = 1/6) *MSH6* – 83% (n = 5/6) *MLH1* & *PMS2* 100% (n=5/5) *MLH1*-p-Hm (+)

dMMR—mismatch repair deficient; IHC—immunohistochemistry; *MLH1*-p-Hm (+): *MLH1* promoter hypermethylation positive; *MLH1*-p-Hm (−): *MLH1* promoter hypermethylation negative.

**Table 4:** Eligible patients for cancer genetic counseling referral, frequency of referral of eligible patients to cancer genetic counseling and frequency of attendance of referred and eligible patients to cancer genetic counseling.

	Frequency of Eligible Patient Referral								Pairwise Comparisons		
	Overall Cohort		Pre-Pandemic (2018–2019)		Early-Pandemic (2020–2021)		Late-Pandemic (2022)		Early- Vs. Pre-Pandemic (Ref)	Late- Vs. Pre-Pandemic (Ref)	Late- Vs. Early-Pandemic (Ref)
	n	%	n	%	n	%	n	%	*p*-Value		
Total number of patients in each cohort	342	100	152	44	139	41	51	15			
^1,2^ Eligible for CGC referral	80	23 (n = 80/342)	37	24 (n = 37/152)	35	25 (n = 35/139)	8	16 (n = 8/51)	0.87	0.20	0.17
^1^ Received CGC referral if eligible for referral	52	65 (n = 52/80)	22	60 (n = 22/37)	25	71 (n = 25/35)	5	63 (n = 5/8)	0.29	0.87	0.62
^1^ Attended CGC if referred and eligible	37	71 (n = 37/52)	17	77 (n = 17/22)	16	64 (n = 16/25)	4	80 (n = 4/5)	0.32	0.90	0.50

CGC—cancer genetic counseling. ^1^ Tested with chi-square test of independence. ^2^ Eligibility for referral to CGC based on (1) mismatch repair deficiency (dMMR) (i.e., absent IHC/MSI-High); (2) age < 50 years of age at the time of diagnosis; (3) absence of *MLH1* promoter hypermethylation; and (4) absence of a previous hereditary cancer syndrome diagnosis.

**Table 5:** Reasons for patients eligible and referred for cancer genetic counseling not attending.

	Pre-Pandemic (2018–2019) (n = 5)	Early-Pandemic (2020–2021) (n = 9)	Late-Pandemic (2022) (n = 1)
Did not attend CGC	– 60% (n = 3/5) inability of schedulers to reach – 20% (n = 1/5) referral expired – 20% (n = 1/5) no documentation	– 44% (n = 4/9) inability of schedulers to reach – 56% (n = 5/9) unwilling	– 100% (n = 1/1) inability of schedulers to reach

CGC—cancer genetic counseling.

## Data Availability

The datasets presented in this article are not readily available because they are identifiable institutional data.

## Appendix A

**Table A1:** Predictors of referral in each time period.

Predictors of Referral Among Patients Eligible for Referral			
*p*-Value ^1,2^			
	Pre-Pandemic (2018–2019)	Early-Pandemic (2020–2021)	Late-Pandemic (2022)
^1^ Age in years (mean)	0.33	0.13	0.26
^2^ EO	0.58	0.33	0.61
^2^ Diagnosis	0.68	0.32	0.62
Rectal cancer	-	-	-
Colon cancer	-	-	-
^2^ Sex	0.55	0.90	0.82
Male (ref)	-	-	-
Female	-	-	-
^2^ Race	0.45	0.90	0.12
White (ref)	-	-	-
African American	-	-	-
^3^ Other	-	-	-
^2^ MMR status	0.68	0.55	0.51
pMMR	-	-	-
dMMR	-	-	-

EO—early age of onset colorectal cancer status, defined as age < 50 years at diagnosis; MMR—mismatch repair; pMMR—mismatch repair proficient; dMMR—mismatch repair deficient; ^1^ tested with chi-square test of independence; alpha = 0.05; ^2^ tested with Wilcoxon rank-sum test; alpha = 0.05; ^3^ Asian, American Indian, Other Unknown

**Table A2:** Predictors of attendance if referred and eligible.

Predictors of Attendance Among Patients Eligible for and Receiving Referral			
*p*-Value ^1,2^			
	Pre-Pandemic (2018–2019)	Early-Pandemic (2020-2021)	Late-Pandemic (2022)
**^1^ Age in years (mean)**	0.72	0.52	0.79
**^2^ EO**	0.41	0.43	0.54
**^2^ Diagnosis**	0.78	0.92	0.61
Rectal cancer	-	-	-
Colon cancer	-	-	-
**^2^ Sex**	0.87	0.92	1.0
Male (ref)	-	-	-
Female	-	-	-
**^2^ Race**	0.48	0.15	0.39
White (ref)	-	-	-
African American	-	-	-
^3^ Other	-	-	-
**^2^ MMR status**	0.70	0.51	0.48
pMMR	-	-	-
dMMR	-	-	-

EO—early age of onset colorectal cancer status, defined as age < 50 years at diagnosis; MMR—mismatch repair; pMMR—mismatch repair proficient; dMMR—mismatch repair deficient; ^1^ tested with chi-square test of independence; alpha = 0.05; ^2^ tested with Wilcoxon rank sum test; alpha = 0.05; ^3^ Asian, American Indian, Other Unknown
